# Chinese Immigrant Wealth: Heterogeneity in Adaptation

**DOI:** 10.1371/journal.pone.0168043

**Published:** 2016-12-15

**Authors:** Lisa A. Keister, Jody Agius Vallejo, Brian Aronson

**Affiliations:** 1 Duke University, Department of Sociology, Durham, North Carolina, United States of America; 2 University of Southern California, Department of Sociology, Los Angeles, California, United States of America; Universidad Veracruzana, MEXICO

## Abstract

Chinese immigrants are a diverse and growing group whose members provide a unique opportunity to examine within-immigrant group differences in adaptation. In this paper, we move beyond thinking of national-origin groups as homogenous and study variation among Chinese immigrants in wealth ownership, a critical indicator of adaptation that attracts relatively little attention in the immigration literature. We develop an analytical approach that considers national origin, tenure in the U.S., and age to examine heterogeneity in economic adaptation among the immigrant generation. Our results show that variations among Chinese immigrants explain within-group differences in net worth, asset ownership, and debt. These differences also account for important variation between Chinese immigrants, natives, and other immigrant groups and provide important, new insight into the processes that lead to immigrant adaptation and long-term class stability.

## Introduction

Chinese immigrants to the United States are a large and growing group, and a recent influx has drawn renewed attention to a relatively small segment of the total Chinese community who have high socioeconomic status (SES) [1–3]. Yet there is a long history of Chinese immigration that has created a diverse population whose experiences offer a rare opportunity to understand contemporary modes of immigrant incorporation. Immigrants from Mainland China, Hong Kong, and Taiwan are, indeed, noteworthy for their high median levels of achievement, a result of hyperselectivity on education and income [4, 5]; however, there is considerable within-group diversity that reflects both individual differences and the historically-unique conditions that were salient in the home and host countries at the time of migration. Looking past the medians, it becomes clear that Chinese immigrants vary dramatically on many traits that are associated with incorporation such as national origin, U.S. tenure, and life course state. Although it is well-established that each of these characteristics facilitates post-migration attainment [2, 6–8], scholars have increasingly called for more exploration of within-immigrant group variation on attributes such as these to adjudicate disparate findings about incorporation [9–12]. Because contemporary Chinese immigrants are the largest and most internally-diverse portion of the Chinese origin population, they provide a unique and important opportunity to answer this call.

We take this challenge seriously and study variations among Chinese immigrants on a critical, but often overlooked, dimension of adaptation: wealth ownership. Wealth, or assets less debts, is an important measure of immigrant economic adaptation for households at all levels of the distribution, and it can be particularly important for immigrants. Owning assets—such as the family home, a business, and financial assets—provides economic security, contributes to adaptation, and facilitates long-term class stability that enables second- and later-generation security and mobility. Debt levels are also revealing: some debt indicates incorporation into the host-country financial system and the potential for future investing (i.e., through business and real estate), while excess debt can signal financial insecurity. It is likely that many Chinese immigrants have high wealth given their relatively high educations and incomes, but the varied life histories and significant diversity among Chinese immigrants on many measures suggests that there is likely to be considerable—and meaningful—heterogeneity on wealth ownership. Immigration research has shown how education affects the adaptation of immigrants and their descendants [5, 13], but immigrant wealth is only beginning to attract research attention. There have been important efforts to document general patterns in Asian wealth [8, 14, 15], but this work looks broadly at Chinese immigrants. The extremes of wealth inequality within Asia, including China, have even brought attention to related issues of inequality in the popular press [16]. We contribute to this discussion by exploring how within-group variations are associated with patterns of asset and debt ownership and, thus, adaptation.

To accomplish this, we first identify ideas from the immigration and wealth literatures that are useful for understanding patterns of wealth ownership among Chinese immigrants. We then address how variations in the historical, economic, and social conditions surrounding both emigration and immigration are likely to contribute to wealth accumulation for Chinese immigrants from Mainland China (MC) and Hong Kong/Taiwan (HK-T). Next, we develop an analytical approach that considers national origin, tenure in the U.S., and age to examine heterogeneity in economic adaptation among the immigrant generation. We study these ideas using the Survey of Income and Program Participation (SIPP), a large-scale nationally-representative data source that contains rich information detail on both country of birth and wealth. The majority of research examining immigrant adaptation concentrates on the second and later generations, but, we take a step back and focus on the immigrant generation because immigrants comprise the bulk of the Chinese population in the United States and because Chinese immigrants remain the largest group of new arrivals to the U.S. These factors necessitate a focused analysis of the first generation to understand the integration of the second generation. We conclude by addressing how varied wealth ownership patterns among Chinese immigrants might influence broader patterns of Chinese American adaptation and inequality in the U.S. and globally.

### Chinese Immigrant Incorporation

Chinese immigration to the U.S. dates to the mid-1800s when migrants were recruited as low-skilled laborers. It increased dramatically after 1965 when exclusionary immigration laws enacted in the late 1800’s that banned Chinese from entering the U.S. were changed with the Immigration and Nationality Act, legislation that created two legal entry pathways: high skill and family reunification [8, 17]. In 2009, Asians surpassed Latinos as the fastest-growing segment of the foreign-born U.S. population [1, 2], and much of this change can be attributed to the Chinese. Chinese Americans are the largest Asian subgroup, at 23%, and 76% of Chinese Americans are foreign-born [1–3]. In addition to their size, contemporary Chinese immigrants to the U.S. are among the most internally diverse group, which makes them particularly important in the study of immigrant adaptation.

There has been intense debate in the immigration literature regarding the degree to which immigrants adapt in the host country; these ideas have been assessed in detail elsewhere [18–21], but a brief summary is useful for understanding Chinese immigrant wealth. Three perspectives dominate this literature. First, segmented assimilation scholars propose that the children of immigrants (second generation) with high SES parents who arrive in the host country legally and for whom the context of reception is positive will also be high SES and will have children (third generation) who approximate the status of the white mainstream. Those with low SES parents who arrive without authorization and under less-welcoming political and economic conditions are likely to become part of a racialized underclass where manual labor jobs, deviant lifestyles, and low income are the norm [19, 22–24]. Second, mainstream assimilation scholars agree that family background, legal status, and context of reception matter; but they argue that class boundaries are more fluid than segmented assimilation suggests [9, 25, 26]. Research in this tradition proposes that immigrants occupy a large number of positions that reflect the complex interplay among background, personal, and contextual influences [9–11].

Third, delayed assimilation scholars agree that there are a large number of positions open to immigrants and their offspring, but they emphasize that parental human capital, the most critical of which is parental education, and immigrant entry status are critical to long-term well-being [13, 27, 28]. Scholars in this tradition discuss a transition phase in the incorporation process for groups that enter without legal status that has the potential to delay assimilation; the second generation is likely to be largely successful in integrating into American society, but some individuals and groups, especially those whose parents lack education and legal status, will experience delayed assimilation. That is, it may take several generations to converge to the middle-class mean. [13, 26]. These perspectives view immigrant incorporation into the mainstream as a process that occurs over time where the descendants of immigrants eventually approximate the status of the white middle-class mean. However, today’s immigrants do not always begin in the lower, or even middle, tiers of the class structure and progress closer to the mean with each successive generation. If conditions of exit and reception are positive and combine with an immigrant stream that is selected on multiple dimensions beyond education, some immigrants and their children may exhibit what we refer to as a pattern of accelerated economic integration, where they, and their descendants, outperform the economic status of long-settled whites, the typical reference group by which integration is measured.

Debates in this literature have been extensive, but much of the immigration literature provides either overly optimistic [2, 29, 30] or pessimistic [19, 31] accounts of the prospects of immigrant groups. Part of the challenge has been a simultaneous focus on multiple immigrant groups and various interacting behaviors and conditions that are associated with incorporation. Studying intra-group heterogeneity—that is, narrowing in and exploring how background, demographic, and contextual traits that vary across members of the same immigrant group relate to attainment—has the potential to adjudicate among some of the complex issues that underlie these debates [9, 12]. An added challenge, however, is that many immigrant groups are relatively homogenous because selection of individuals and families into immigration combine with historic events and socioeconomic conditions in the sending and host countries to reduce differences within particular groups. Mexican immigrants have been studied extensively in the immigration literature in part because they are such a heterogeneous group [9, 20, 32, 33].

Chinese immigrants have attracted attention in this literature [8, 34–36], but neither their within-group differences nor their wealth have been explored in detail. Chinese immigrants to the United States are somewhat unique in their long history of immigration and the conditions in both the sending and host countries that surrounding waves of immigration. There are also important differences in the selection processes that influence individual and family decisions to immigrate given that Chinese come from Mainland China, Hong Kong, and Taiwan. Chinese immigrants and their descendants are often valorized by media, politicians, and scholars as model minorities whose high levels of educational attainment are reflective of Asian cultural values [37]. However, scholars have recently underscored that Chinese Americans’ advantaged structural position, regardless of class background, results from their educational selectivity [4], and U.S. immigration laws favoring the entry of the highly educated [5, 8]. We contribute to these debates and move beyond the traditional emphasis on educational attainment by studying variations among Chinese immigrants in wealth ownership.

### Wealth and Incorporation

Wealth is a critical indicator of well-being for both those who own it and those who do not [38–40]. For the wealthy, assets can generate interest and dividends that create additional wealth if reinvested; but even a small amount of savings or home equity can have enormous advantages. Assets can improve education for current and future generations, create a safe and pleasant living environment, make retirement secure, and provide an essential financial buffer against income interruptions, medical emergencies, and other financial shocks. It is understudied in the immigration literature, but wealth is an important indicator of adaptation because it signals economic well-being for its owner, resources available to families, and long-term class stability. Savings, and inheritances, also create a buffer against third-generation decline, the downward or stagnated mobility that threatens well-being across immigrant generations [33]. Moreover, immigrants consider socioeconomic position an important component of status, and wealth closely approximates their conceptions of attainment [10, 22, 41]. Understanding household-level immigrant wealth ownership is also important for understanding aggregate patterns of wealth inequality for immigrants and for the U.S. more generally. After all, understanding how households save is an important first step in understanding broader patterns of resource distribution across households.

Two approaches from the wealth inequalities literature are useful for understanding Chinese immigrant wealth. First, the status attainment model is fundamental for understanding how background and individual traits are associated with adult outcomes [42–44] and is particularly useful for studying wealth which is inherently cumulative [45, 46]. A weakness of this model is its failure to capture the effect of key events on long-term outcomes [47]. To resolve this, sociologists draw on a second approach, the life course tradition, which identifies turning points for individuals and cohorts and common trajectories that result from those turning points [48]. The life course approach is distinct from the similarly-named life cycle hypothesis from economics which proposes that people spread consumption and saving over their lifetimes. For accumulated wealth, this implies that people save until they retire and dissave after; that is, wealth should grow until retirement then declines [49]. Empirical evidence shows considerable nuance across households; for example, for some households, wealth continues to grow after retirement because of uncertainty about the timing of death and the bequest motive [48]. These traditions are rarely invoked explicitly in the immigration literature, but they underlie work that explores generational processes [23, 50] and historic events [9, 51] in immigrant adaptation.

### Heterogeneity in Adaptation

The immigration and wealth literatures suggest three traits on which Chinese immigrants vary that are likely to shape their wealth: national origin, U.S. tenure, and age.

#### National Origin

National origin is well-known to be associated with incorporation, but there are few opportunities to compare immigrants who share ethnicity and many components of culture, national history, and even politics but still originate from unique places. Chinese immigrants from Mainland China, Hong Kong, and Taiwan provide such an opportunity. Complete overviews of the histories, economies, and social structures of these three places are available elsewhere (see, for example, Chow 2015, Naughton, 2006, Hsiao and Hsiao 2015, Kim 1999), but some details are important to mention. For example, Mainland China (i.e., the People’s Republic of China), the source of the largest numbers of current immigrants to the U.S., was now-famously disconnected from most other countries for 30 years following the 1949 communist revolution and experienced little economic growth until Deng Xiaoping instituted the massive reforms in 1979 that spawned more than 36 years of subsequent growth and concurrent high levels of inequality [16]. By contrast, Hong Kong became a British colony with a 99-year lease in 1898 following the First Opium War; the territory was returned to the People’s Republic of China in 1997 and has retained a high degree of autonomy under the Hong Kong Basic Law. Taiwan (i.e., the Republic of China) separated from Mainland China in 1949 following the Chinese war when the Communist Party of China took control of the mainland. During the second half of the twentieth century, both Taiwan and Hong Kong experienced rapid economic growth and industrialization [52–54]. Both surpassed Mainland China in their levels of growth by the 1970s, and both were more open to emigration and other exchange with foreign countries. Consequently, more people migrated from Taiwan and Hong Kong to the U.S. than from Mainland China, and given their countries’ greater contact with the west, migrants arrived with human and social capital that were conducive to material success in the west [8]. However, it is likely that cross-region differences will be evident in the wealth ownership of immigrants to the U.S.

In addition to within-group differences among Chinese immigrants, there are likely to be differences between Chinese immigrants and other immigrant groups—including high-wealth immigrant groups such as Asian Indians and Europeans—and long-settled black and white Americans. Chinese immigrants vary on important dimensions, including class and national origin, but Chinese immigrants to the U.S. are a hyperselected immigrant group, which means that they have higher median levels of education than those left behind *and* those in the receiving nation (Lee and Zhou 2015). China is the primary source of foreign students to American universities and represents the second-largest segment of entry visas for work, which disproportionately prioritize high-skills [55]. Despite important differences in the emigration contexts, mainlanders, especially the early wave, tend to be college students who entered the U.S. on student visas, whereas Taiwan and Hong Kong are skilled-export nations with the U.S. being the primary destination [8]. Moreover, China, Hong Kong, and Taiwan have higher savings rates than the U.S. [56], with especially high rates in China where saving occurs at 34–54% of GDP, a phenomena referred to as the “Chinese Savings Puzzle” [57, 58].

#### U.S. Tenure

The association between host country tenure and attainment is a source of much of the controversy underlying in the immigration literature, and unique tenure-wealth patterns for Chinese immigrants to the U.S. have the potential to shed some light on how tenure operates. The number of years an immigrant has spent in the host country is an indicator of the amount of time the person has had to adapt to conditions in the new context; this suggests that tenure should be strongly and positively associated with attainment. However, debates emerge in part because some groups do not attain expected levels of income, education, and wealth after time in the host country. This literature tends to focus on the role that host country conditions—the context of reception—play in facilitating attainment, but tenure since immigration provides critical information about the context under which ***both*** emigration ***and*** immigration occurred. Indeed, the context under which immigrants leave the home country—the emigration context—is arguably critical to their long-term well-being.

We propose that it is useful to think of three broad waves that represent changes in both home and host country context. The dates we propose are approximations that reflect continually changing conditions at home and in the U.S. First, those who migrated more than 25 years ago (between 1965 and 1989) from Mainland China left a country that was still developing, urbanizing, industrializing, and otherwise evolving economically. Economic growth began to accelerate in the 1980s on Mainland China, but development was still limited [59–61]; migration to the U.S. grew, but, in contrast to those from Hong Kong and Taiwan, those who migrated had little initial exposure to the types of human, social, and cultural capital that are associated with success in the west. However, Mainland migrants entered under the newly-enacted visa structure, which privileged family reunification and high-skilled applicants, especially university-bound students, who tended to stay in the U.S. upon graduating and generally attain high-skilled jobs (Zhou 2009).

U.S. policy favoring Chinese immigrants at the end of this time period might also have supported wealth accumulation among Chinese immigrants, especially those who migrated in the 1980’s. The U.S. government granted special privileges to Chinese immigrants, both those with visas and those without, in response to the Tiananmen Square protests in China that culminated in a violent massacre of Chinese students in the summer of 1989. Chinese nationals were allowed to remain in the U.S. and granted authorization to work, and eventually, more than fifty thousand Chinese immigrants who were in the U.S. at the time of Tiananmen Massacre received permanent residency under the 1992 Chinese Student Protection Act. These government actions created a context that provided highly educated Chinese immigrants the benefits of legal residency, which allowed them to work in high-skilled jobs without having to obtain an employer sponsor, and subsequently exerted a significant and positive impact on their employment and earnings outcomes [62].

Taiwan and Hong Kong were more developed than Mainland China prior to 1989, but these two countries have also changed considerably in ways that are likely to make early waves of immigrants unique [53, 63]. Hong Kong was still developing and industrializing prior to 1989, yet it operated as an independent nation state with a social structure and culture that borrowed heavily from Great Britain. Taiwan was wholly independent, although it was still developing and industrializing as well. The context of reception in the U.S. was also developing 25 years ago. Of course, Chinese American communities have a long history in the United States, and their unique patterns of assisting new immigrants have been well-documented [8]; but the modern Chinese communities that are often instrumental in providing economic and social support to new immigrants were just beginning to expand and Chinese communities were still quite rare in other parts of the U.S. prior to 1989.

A second wave, those who migrated between 11 and 25 years ago (1990–2004), would have emigrated from and to contexts that were markedly different from earlier waves of immigrants. The 1990s and early 2000s saw notable changes in all three regions that are likely to be reflected in the experiences of those migrating during this era. Mainland China continued to grow and expand rapidly [64, 65]; there were dramatic changes in the country’s social structure, markets developed rapidly, and urbanization increased. Immigration to the United States also increased; moreover, those who migrated were more accustomed to the educational, social, cultural, and economic structures that they encountered upon immigration. Changes also continued in Hong Kong, where economic growth has not stagnated, but it has slowed relative to earlier decades. Moreover, return to Chinese control in 1997 meant closer ties with the Chinese central government; the official policy of “one country, two systems” means that Hong Kong retained autonomy, but the social, economic, and political connections between the two are clear. Economic growth in Taiwan also slowed, but again did not stagnate, and the country underwent considerable democratic reforms in the 1990s that culminated in the first direct presidential election in the nation’s history. At the same time, Chinese communities in the U.S. continued to expand both in size and number; by the early 2000s, it was common to find relatively well-established Chinese communities in cities across the United States offering immigrants the social, economic, and cultural assistance that can facilitate the transition process.

A third wave, roughly since 2004, includes immigrants who have left relatively well-developed home countries and have arrived to equally well-developed Chinese communities in the U.S. Economic growth on Mainland China has largely continued, and concurrent social and cultural changes have produced waves of migrants who arrive with experiences and capital that makes them better positioned than earlier waves of immigrants to adapt to the U.S. context. Similarly, immigrants from Hong Kong and Taiwan arrive with the human, social, and cultural capital that facilitates adaptation into the U.S. mainstream that was perhaps more difficult for early waves from these regions. The growth and development of Chinese communities in the U.S. has also continued creating an arrival context that is both welcoming and encouraging. Some large Chinese communities such as Monterey Park (and surrounding communities) near Los Angeles have attracted attention in both the popular press and from academics, but Chinese communities have expanded in cities across the U.S.

#### Age and Life Experience

Age and life experience have been central to—and at times controversial components of—explanations of wealth ownership. Attainment and life course scholars assume a relatively straight line wealth accumulation pattern over time, although life course research implies that individually-determined turning points may alter an accumulation trajectory [66, 67]. For instance, divorce may reduce the rate of accumulation for both parties). By contrast, life cycle research implies that individuals will accumulate wealth through retirement and then dissave. Chinese immigrants are likely to have unique wealth ownership patterns that may appear consistent with the life cycle hypothesis from economics but that, in reality, underscore the importance of the life course approach from sociology. That is, when Chinese immigrants are viewed as a collective, their wealth is likely to increase with age until retirement and then decline as the life cycle hypothesis proposes. Yet this broad pattern masks an underlying demographic pattern: the age structure of Chinese immigrants is unusual, including disproportionate numbers of young professionals and their older parents. The markedly different life course experiences of these groups, which reflect the conditions of emigration discussed above, is likely to result in high wealth for younger people and low wealth for their older parents.

### Expectations

These trends and ideas suggest several specific patterns. First, Hong Kong and Taiwanese (HK-T) immigrants are likely to have high wealth compared to Mainland Chinese (MC) immigrants and other high-wealth groups reflecting the earlier economic development of their home countries and hyperselection into immigration. That is:

H1A: HK-T immigrants have higher net worth than MC immigrants, native whites and blacks, and other immigrant groups.

By contrast, MC immigrants are likely to have lower overall wealth reflecting Mainland’s slight lag in development, but selection and age effects are still likely to give them an advantage. Specifically, we expect that:

H1B: MC immigrants have lower net worth than HK-T immigrants, and native whites, but they have higher net worth than Latino immigrants and native blacks.

Differences among Chinese immigrants are also likely to be reflected in differences in the components of net worth, or total assets owned and debts held. The propensity to have any assets and the value of assets owned reflect access to saving instruments as well as a group’s propensity to save, invest, and start businesses. Similarly, debts or liabilities indicate integration into the financial system as well as a propensity to invest, particularly when the debt is used to purchase real estate or start a business. Of course, excess debt can signal financial strain that indicate potential long-term challenges for households and whole groups. That is:

H2A: HK-T immigrants have high propensities to have any assets or debts and, for those with assets or debts, are likely to have higher total values than MC immigrants, native whites, native blacks and other immigrant groups.

Despite their relatively low levels of net worth, MC immigrants are likely to have high rates of asset ownership, high saving rates, education and occupation selection, and median age. For those who own assets, it is also likely that the value will be relatively high. However, because MC immigrants have lower starting points for accumulating given home country levels of development, the value of their total assets is likely to be lower than for HK-T immigrants. Less connection to the U.S. financial system, however, suggests that a different pattern is likely for debt. In particular, HK-T immigrants are likely to have relatively high rates of debt consistent with business startup and the higher rates of household debt in the home country. By contrast, MC immigrants are likely to have relatively low propensities to have any debt, consistent with home country trends. Specifically:

H2B: MC Chinese immigrants have high propensities to have any assets; and for those with assets, the values are higher than for white and black natives but not as high as those of HK-T immigrants.

H2C: MC immigrants are less likely than HK-T immigrants and native whites to have any debts, and debt levels for those with any liabilities are likely to be lower than for these other groups.

Of course, wealth ownership is likely to increase with tenure in the U.S. for all immigrant groups, including Chinese immigrants; but we expect that time in the host country will narrow the gap among Chinese immigrants and between MC immigrants and native whites. That is:

H3: As tenure in the U.S. increases, the wealth of MC immigrants will become more similar to that of HK-T immigrants and native whites.

The association between age and wealth is likely to be unique for Chinese immigrants, reflecting education selection and age patterns. Specifically, we expect that:

H4: There is an inverted U-shaped relationship between age and wealth for all Chinese immigrants, and the wealth of older Chinese immigrants is lower than that of native whites.

### Data

Our data are from the SIPP, a multipanel, nationally-representative survey of 14,000–36,700 U.S. households interviewed every 2–4 years. The SIPP data are the best available data for our purposes because contain both detailed country of birth information and detailed wealth information. The SIPP wealth modules include information on both assets and debts and are comparable for most households to other surveys of household wealth (Hao 2007, Keister 2014). Because the SIPP is nationally-representative—a unique feature for data on immigrants—and has a large sample, we are able to study Chinese immigrants living across the U.S. The large SIPP sample contains details on respondent country of origin, including within-Asia origin, for immigrants which allows us to compare Mainland Chinese immigrants to those from Hong Kong and Taiwan. Our sample includes 448 respondents who were born in Mainland China and 276 who were born in Hong Kong or Taiwan. There are no other large, nationally-representative data sets that include both detailed ethnicity and detailed wealth information. Moreover, the SIPP includes multiple years of data and data on a wide range of other topics that are useful for isolating the effect of our key variables on wealth ownership. We use data from 1996, 2001, and 2004, the most recent waves in which detailed country of origin information are available. We also verified our findings using non-public 2008 SIPP data at a Census Data Research Center. We do not report 2008 results per Census confidentiality requirements, but the findings are consistent with those that we present here.

As is the case with any dataset, the SIPP data are not perfect. Because the SIPP removed its nativity measure from the public data in 2008, we do not use the most current survey year in the results displayed. However, we obtained access to the non-public SIPP data and ran our models including the 2008 data; adding the additional survey year did not change our results. The Census prevents us from including the 2008 models to protect confidentiality. We would also prefer to have data before and after the 2007–09 recession, but to our knowledge, no nationally-representative data including ethnicity and wealth exist. Ideally, the data would contain longitudinal information on respondents so that we could study accumulation patterns over time for the same respondents, but the SIPP contains only cross sections. We are careful not to discuss accumulation processes and to focus on wealth states instead. In addition, the SIPP includes only citizens and legal residents, and it categorizes the native-born by panethnicity. For these reasons, we are unable to address how legal status mediates the processes we study; we are also careful to compare our focal groups only to natives and other immigrants. Perhaps the most significant drawback is the relatively small sample sizes for Hong Kong and Taiwanese immigrants, and we group these immigrants to ensure appropriate degrees of freedom. Finally, the SIPP data do not have an oversample of high-wealth households which means that we are unable to calculate measures such as the Gini coefficient.

Comparing immigrants from Mainland China to those from HK-T allows us to hold constant some elements of culture and ethnicity while allowing other important factors to vary given that the conditions and context under which people emigrate from these markedly different places are likely to be associated with post-immigration well-being. Moreover, there are important similarities between Hong Kong and Taiwan including recent changes in economic development, relationship to the Chinese government, and geographic proximity to the mainland. Of course, we do not intend to downplay the extraordinary difference between Hong Kong and Taiwan in their histories, social structures, economic systems, and political situations; unfortunately, however, we are unable to explore those differences in depth in this paper.

### Variables

We measure wealth with three variables. **Net worth** is the sum of the current market value of total household assets less the value of total debts. **Total assets** are stocks, bonds, mutual fund shares, retirement accounts, checking and savings accounts, and real estate. **Debts** include mortgages, consumer loans, student loans, vehicle loans, and other debt. All values are adjusted to 2013 dollars using the Consumer Price Index (CPI). These measures provide a comprehensive portrait of the accumulated savings and corresponding liabilities of a household. Although the SIPP collect information on all individuals in a household, wealth is appropriately included as a household-level variable and weighted to be representative of U.S. households.

**National origin** is country of birth. We compare Chinese immigrants from Mainland China to those from Hong Kong and Taiwan, and we compare both of these groups to native Asians, Asian immigrants, Latino immigrants, native whites, native African Americans. We focus on the foreign born because our data prevent us from making detailed comparisons with native-born Chinese Americans and because we are concerned with factors related to within-group heterogeneity among immigrants. Comparing Chinese immigrants to native whites and African Americans is reasonable and important because both of these native groups have well-documented wealth ownership patterns and well-established positions in the U.S. status hierarchy that provide a baseline for understanding Chinese immigrants.

**Tenure in the U.S.** is years from the time of immigration. We use three tenure categories to correspond to our expectations: 0–10, 11–24, and 25 or more years. **Age** is the current age of the household head measured as three categories: 25–39, 40–54, and 55 or more years.

We control for **education** using five categories: non-high school graduates, high school graduates, some college, college degree, and advanced degree. Using a continuous measure of years of education does not change the results, and these categories represent the patterns in the data most accurately. **Income** is total annual household income adjusted to 2004 dollars using the CPI. We also control for having a female household head, the presence of children, and marital status. Using other family structure indicators (e.g., additional marital status categories) did not improve model fit. We control survey year to hold constant unusual events that affect all households (e.g., financial booms). We control **region** of residence with five categories: midwest, northeast, south, west, and other (e.g., U.S. territories).

## Results

### Demographic Heterogeneity

Descriptive statistics (Table 1) demonstrate heterogeneity in Chinese immigrants’ demographic characteristics, including on national origin, U.S. tenure, and age. Consistent with the recent opening of emigration from Mainland China, 43% of MC immigrants have lived in the U.S. 10 years or less, compared to just over a quarter of HK-T immigrants. By contrast, 43% of HK-T immigrants have lived in the U.S. for nearly 25 years, compared to 30% of MC immigrants. MC immigrants are older than HK-T immigrants, and there is considerable variation in educational attainment among Chinese immigrants. More than half of MC immigrants have a college degree or more education, compared to just over two thirds of HK-T immigrants. At the other end of the educational spectrum, 21% of MC immigrants have less than a high school degree, compared to only 6% of the HK-T immigrants. Notably, education levels are higher for Chinese immigrants, regardless of national origin, than for native-born Americans and other immigrant groups, except Asian Indians.

**Table 1:** Chinese Immigrants: Heterogeneity on Demographic Traits.

		Tenure in U.S. (years)				Education (%)					
Race/National Origin	n	0–10	11–24	25+	Age	Less than HS	HS Graduate	Some College	College	Advanced Degree	Female Head
Mainland Chinese	327	0.43	0.30	0.23	49.95	0.21	0.19	0.07	0.25	0.29	0.20
HK-Taiwan	150	0.27	0.43	0.29	45.14	0.06	0.16	0.10	0.37	0.31	0.19
Asian Indian	330	0.52	0.29	0.16	40.43	0.03	0.07	0.07	0.39	0.44	0.07
Other Asian	1530	0.26	0.37	0.33	45.95	0.12	0.20	0.13	0.41	0.14	0.20
Native Asian	1283				47.45	0.09	0.22	0.20	0.40	0.09	0.28
Mexican	2371	0.30	0.36	0.32	42.35	0.58	0.22	0.08	0.10	0.02	0.20
Cuban	329	0.16	0.18	0.62	55.64	0.27	0.28	0.15	0.25	0.05	0.26
Other Latino	1968	0.28	0.32	0.37	47.14	0.22	0.27	0.14	0.27	0.09	0.31
Native Latino	3537				45.33	0.27	0.29	0.18	0.22	0.04	0.30
Other Immigrants	2300	0.24	0.18	0.54	52.29	0.13	0.25	0.14	0.32	0.16	0.27
Native Black	10127				48.85	0.21	0.30	0.19	0.24	0.05	0.49
Native White	63887				52.06	0.11	0.29	0.18	0.32	0.10	0.27

Notes: Data are from the Survey of Income and Program Participation (SIPP).

### National Origin and Wealth

Consistent with our expectations, there is a clear association between national origin and net worth. Table 2 compares the percent of households with positive net worth as well as mean and median net worth by race and national origin. The estimates demonstrate that HK-T immigrants have higher median net worth than MC immigrants: the HK-T median is $259,207, while the MC median is $126,494. The mean, which reflects extremely high net worth households, is even higher for HK-T immigrants ($411,753) than for MC immigrants ($263,434). The estimates in Table 2 are also consistent with our expectation that HK-T immigrants have higher net worth than native whites and other immigrant groups. Table 2 shows that both mean and median net worth for HK-T immigrants surpasses that of all other groups included in the Table. By contrast, MC immigrants have lower net worth than native whites, but their mean and median net worth are both higher than that of native blacks and of the Latino immigrant groups that we include in the Table. Some of this difference is likely a result of selection on education and income, suggesting that multivariate models would be useful to clarify the degree to which national origin and other factors are at play.

**Table 2:** Chinese Immigrant Net Worth.

Race/National Origin	Net Worth > 0	Mean	Median
Mainland Chinese	0.85	$263,434	$126,494
HK-Taiwan	0.93	$411,753	$259,207
Asian Indian	0.87	$326,104	$163,400
Other Asian	0.84	$245,037	$105,275
Native Asian	0.83	$262,691	$118,853
Mexican	0.77	$99,397	$29,921
Cuban	0.82	$180,304	$100,064
Other Latino	0.76	$188,386	$72,286
Native Latino	0.75	$158,960	$60,096
Other Immigrants	0.87	$384,798	$147,713
Native Black	0.70	$105,302	$46,756
Native White	0.90	$318,970	$152,412

Notes: Data are from the Survey of Income and Program Participation (SIPP); estimates are proportion of respondents with net worth greater than zero and mean/median for those with net worth greater than zero; values are in 2013 dollars.

Variations among Chinese immigrants by national origin, U.S. tenure, and age are even more pronounced in the multivariate models shown in Table 3. The coefficient estimates included in this Table are from OLS regression models of total (logged) net worth. Model 1 is a base model that does not include controls and demonstrates the strength of the association between national origin and net worth with only tenure and age held constant. In Model 2, we add controls for education and income to explicitly take immigrant selection into account; and in Model 3, we include all control variables. The national origin differences in wealth among Chinese immigrant groups are clear across these models: HK-T immigrants have net worth that is significantly higher than that of MC immigrants, native whites, and the other immigrant groups included in the table. Cox tests indicate the differences in coefficients across models are significant. That is, the decline in the coefficient values from Model 1 through Model 2 is a significant difference that reflects the relative importance of the control variables. Notably, however, the differences we anticipated in net worth by national origin persist even in the full models, a pattern that has implications for theoretical models of immigrant adaptation. Traditional models assume that immigrants start off behind long-settled native born whites and that adaptation is evident when the second and third generations converge to the white middle-class mean, but the HK-T immigrants we studied have higher levels of wealth than native-born whites when other factors are controlled (Table 3, Model 3). In fact, the first-generation HK-T immigrant median already exceeds the white median, suggesting that HK-T immigrant wealth ownership is more consistent with the accelerated economic adaptation model.

**Table 3:** OLS Regression Models of Total Net Worth (logged).

	Model 1. Base model	Model 2. Add education & income	Model 3. Add all controls
**Constant**	1.03***	-0.22***	-0.19***
	(0.03)	(0.04)	(0.05)
**Race/National Origin**			
Mainland Chinese	2.34***	1.89***	1.50***
	(0.18)	(0.17)	(0.17)
HK-Taiwan	3.63***	2.89***	2.58***
	(0.23)	(0.22)	(0.22)
Asian Indian	3.04***	1.91***	1.48***
	(0.18)	(0.17)	(0.17)
Other Asian	1.95***	1.49***	1.14***
	(0.12)	(0.12)	(0.11)
Native Asian	1.29***	0.90***	0.67***
	(0.09)	(0.08)	(0.08)
Mexican	1.07***	1.52***	1.09***
	(0.11)	(0.11)	(0.11)
Cuban	1.30***	1.21***	0.96***
	(0.17)	(0.16)	(0.16)
Other Latino	1.22***	1.11***	0.87***
	(0.12)	(0.11)	(0.11)
Native Latino	0.59***	0.61***	0.37***
	(0.05)	(0.05)	(0.05)
Native White	1.81***	1.43***	1.18***
	(0.03)	(0.03)	(0.03)
Other Immigrant	2.05***	1.63***	1.36***
	(0.11)	(0.11)	(0.11)
Missing Ethnicity	0.45***	0.27***	0.15***
	(0.04)	(0.04)	(0.04)
**Tenure in US (years)**			
0–10	-1.25***	-1.19***	-1.21***
	(0.11)	(0.10)	(0.10)
11–24	-0.70***	-0.64***	-0.63***
	(0.11)	(0.10)	(0.10)
25+	-0.31**	-0.39***	-0.29**
	(0.10)	(0.10)	(0.09)
**Age (years)**			
40–54	1.26***	1.16***	1.15***
	(0.02)	(0.02)	(0.02)
55+	1.91***	2.47***	2.46***
	(0.02)	(0.02)	(0.02)
**Education**			
HS Graduate		0.72***	0.65***
		(0.03)	(0.03)
Some College		0.86***	0.80***
		(0.03)	(0.03)
College		1.25***	1.17***
		(0.03)	(0.03)
Advanced Degree		1.60***	1.56***
		(0.04)	(0.04)
**Income**			
Annual		0.01***	0.01***
		(0.00)	(0.00)
**Family**			
Female Head			-0.20***
			(0.02)
Children			-0.01
			(0.01)
Married			0.93***
			(0.02)
**Year**			
year 1996			-0.12***
			(0.02)
year 2001			-0.07***
			(0.02)
**Region**			
Midwest			0.05
			(0.02)
Northeast			-0.03
			(0.03)
South			-0.05*
			(0.02)
Other			-0.17
			(0.10)

Notes: Data are from the Survey of Income and Program Participation (SIPP).

Standard errors in parentheses.

*p < .05.

** p < .01.

*** p < .001.

Reference national origin is native blacks, reference tenure is natives, reference education is less than high school, reference year is 2004, reference age is 25–39, reference region is west.

As we anticipated, these differences are evident in the assets and debts held by Chinese immigrants as well. Table 4 decomposes wealth into total assets and debts and shows that Hong Kong and Taiwanese immigrants are highly likely to have any assets or debts and have high values for their holdings relative to Mainland immigrants, native whites, and other immigrants as well. By contrast, Mainland immigrants are highly likely to have some assets and to own more of those assets than native whites and blacks, but their asset ownership is not as high as that of Hong Kong and Taiwanese immigrants. The debts of Mainlander are also unique: Table 4 shows that Mainland immigrants are less likely than Taiwanese and Hong Kong immigrants to have any debts, and their debt levels are lower as well. These differences in asset and debt levels reflect home country differences and opportunities in the U.S. to engage with the financial system, as we anticipated. Perhaps most interesting is that these asset and debt levels suggest a positive trend for long-term class stability for both Taiwanese/Hong Kong immigrants who are already engaged financially and for those from Mainland whose are engaged in asset ownership but not over-burdened with liabilities. We do not include additional regression results to conserve space, but we find that these results persist in multivariate models.

**Table 4:** Wealth Components: Proportions and Median Values for Those with Any.

Race/Nativity	Any Assets	Any Debt	Assets	Debt
Mainland Chinese	0.93	0.61	$225,441	$107,359
HK-Taiwan	0.95	0.77	$407,202	$146,949
Asian Indian	0.98	0.80	$257,634	$125,041
Other Asian	0.95	0.75	$196,477	$104,914
Native Asian	0.97	0.78	$168,544	$58,732
Mexican	0.91	0.65	$59,367	$40,611
Cuban	0.91	0.65	$183,640	$70,448
Other Latino	0.90	0.69	$121,209	$54,505
Native Latino	0.91	0.73	$99,792	$44,390
Other Immigrants	0.96	0.68	$204,898	$71,994
Native Black	0.88	0.67	$73,240	$29,176
Native White	0.98	0.76	$214,276	$71,109

Notes: Data are from the Survey of Income and Program Participation (SIPP); estimates are proportion of respondents with assets or debts greater than zero and median values for those with assets or debts greater than zero; values are in 2013 dollars.

### U.S. Tenure and Wealth

**Fig 1:**
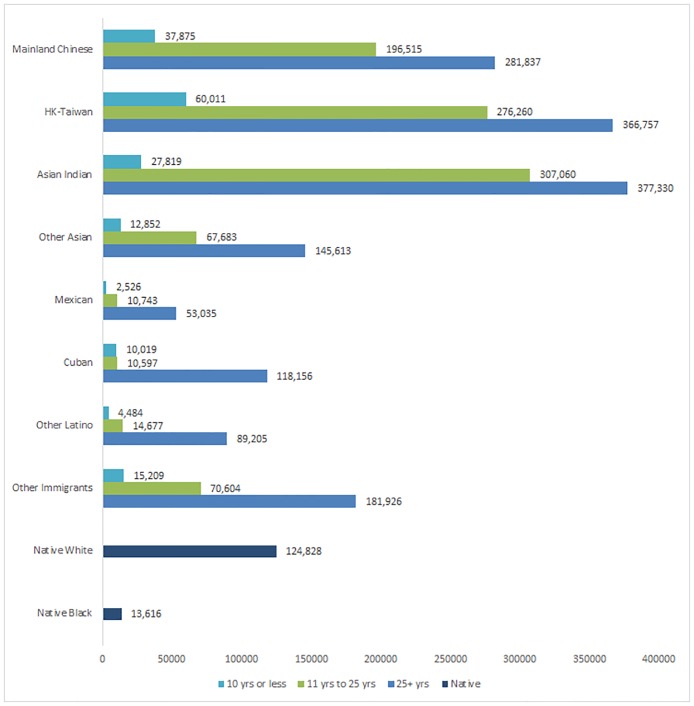
Immigrant Median Net Worth by U.S. Tenure. Data are from the Survey of Income and Program Participation (SIPP); median values for all respondents. Estimates differ from Table 1 which includes medians for those with net worth greater than zero; values are 2013 dollars.

The degree to which U.S. tenure mediates the association between national origin and wealth is evident in Fig 1, a graphical depiction of results from Table 1. Not surprisingly, median net worth increases with U.S. tenure for all immigrants. However, this finding underscores the degree to which Chinese immigrants are unique in their wealth accumulation and highlights the important within-group variation that is central to understanding the attainment of immigrants from Mainland, Hong Kong, and Taiwan. Consistent with our expectations, the wealth of Mainland Chinese immigrants, although still lower at all levels of tenure, begins to approximate the wealth of Hong Kong/Taiwanese immigrants and native whites after they have spent multiple decades in the U.S. Hong Kong and Taiwanese immigrants who have resided in the U.S. for more than twenty-five years have the highest levels of net worth, at $366,757, compared to $281,837 for Chinese immigrants with the same U.S. tenure. Also noteworthy is that the most recent wave of immigrants from Hong Kong and Taiwan has nearly double the wealth of the most recent immigrant cohort from the Mainland. Together, these variations reflect not only the relationship between tenure in the host country and net worth, but also HK-Taiwanese immigrants’ economic head start. In fact, immigrants from HK-Taiwan have higher levels of wealth regardless of tenure in the U.S., compared to those from the Mainland. These variations by tenure and country of origin have implications for adaptation, as long-settled Chinese immigrants have more wealth on which to draw to facilitate their incorporation.

We also anticipated that Chinese immigrants, irrespective of national origin and tenure in the U.S., would exhibit higher levels of net worth when compared to other immigrant groups. Although there is heterogeneity in net worth by their country of origin and tenure, Chinese immigrants have notably high levels of wealth compared to nearly all other immigrant groups with the same U.S. tenure. For example, immigrants from Taiwan-HK who have lived in the U.S. twenty-five years or longer have two-and-half times the wealth of other Asian immigrant groups (except for Asian Indians). The differences with Mexicans are particularly stark. The most recent immigrants from the mainland and HK-T have net worth totaling $37,875 and $60,011 respectively, compared to $2,526 for Mexicans. Among the longest-settled cohort of immigrants, those from HK-T have almost seven times the wealth of Mexicans. These extraordinarily high levels of wealth among the Chinese immigrant generation that only increase with tenure in the U.S. have profound implications for immigrant adaptation. Most critical is that these high levels of wealth entrench economic stability among the immigrant generation which supports an economically advantaged starting position for the children of Chinese immigrants. This wealth might also be available for the second generation as they come of age in the U.S., placing them on an accelerated adaption path relative to other immigrant groups and helping to explain their high levels of SES attainment.

### Age and Wealth

**Fig 2:**
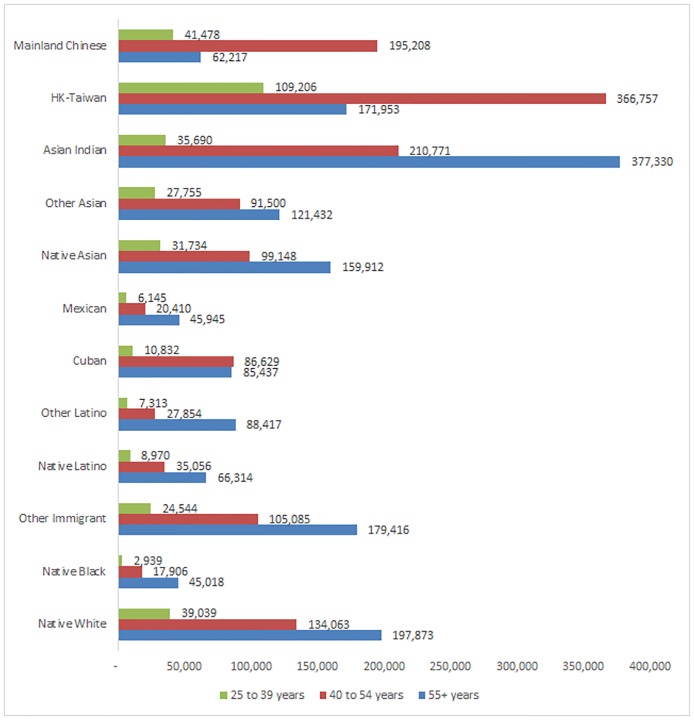
Immigrant Median Net Worth by Age. Data are from the Survey of Income and Program Participation (SIPP); median values for all respondents; values are 2013 dollars.

Age might seem to have a clear connection with wealth, but Chinese immigrants demonstrate that the age-wealth relationship is not obvious. All models in Table 3 include age, and the results are consistent with our expectations: there is an inverted U-shaped relationship between age and wealth for all Chinese immigrants. Fig 2 illustrates the patterns from Table 1. This Table shows that the age-wealth relationship is linear and increasing for most groups, with Chinese being an exception. Among Chinese immigrants, the middle cohort (40–54) has the highest levels of net worth. Mainlanders in the middle-age cohort have three times the median net worth ($195,208) of the oldest Chinese immigrants from the Mainland ($62,217). Similarly, immigrants from HK-Taiwan have more than double the amount of total net worth ($366,757) than the oldest immigrants from their countries ($171,953). We suspect that these differences reflect a trend of older and relatively less advantaged Chinese immigrants migrating to the U.S. to be closer to their adult children, a pattern that reflects the cultural norm of filial piety that has typically characterized intergenerational relationships in Chinese families [68]. Parental immigration is also supported by U.S. immigration laws favoring family reunification. About half of Chinese immigrants to the U.S. receive a green card via family reunification [55] and the parents of American citizens receive priority [69]. Perhaps more importantly, these findings underscore both the variation among Chinese immigrants in adaptation and the degree to which the association between age and adaptation varies across immigrant groups. Given that starting point and life experience are both critical components of adaptation, these patterns suggest that the middle age cohort for both MC and HK-T immigrants are the most advantaged. These differences might ultimately translate into varying patterns of educational and occupational attainment of second-generation Chinese Americans depending on parental national origin, their parents’ tenure in the U.S., and parental age cohort.

## Discussion and Conclusions

Chinese immigrants to the U.S. are a large, diverse group who provide a unique opportunity to understand patterns of immigrant incorporation. We contribute to the literature in two key ways. Methodologically, we employ an analytical approach that considers background variables and historical events—measured via national origin, tenure in the U.S., and age—to analyze heterogeneity in economic adaptation among the immigrant generation. This analysis allows for greater explanatory power when untangling deviating adaptation paths within and between groups, especially among groups comprised of both longer-settled immigrants and new arrivals, and groups who also hail from varying regional contexts. We draw on the high levels of within-group diversity on national origin, U.S. tenure, and age for Chinese immigrants to understand why some immigrants accumulate wealth—an important measure of adaptation—more quickly than others. In doing so, we answer a call from the immigration literature to explore within-group heterogeneity in order to adjudicate competing explanations, and reformulate theories, about incorporation[9–11]. We proposed that both the context of emigration—the conditions under which migrants leave the home country—and the context of immigration—the more commonly-studied conditions encountered upon arrival in the host country will condition the ultimate incorporation of Chinese immigrants. Thus, we anticipated finding important differences in wealth ownership by national origin (i.e., between MC and HK-T immigrants). We also anticipated that U.S. tenure and age would be important dimensions on which Chinese immigrants vary. Our analyses of SIPP data showed that HK-T immigrants had, indeed, accumulated higher levels of net worth than MC immigrants; consistent with our expectations, hyperselection of HK-T immigrants appeared to interact with relatively high levels of development in the home country to push the wealth of HK-T immigrants passed native whites and other immigrant groups. Similarly, a less-developed context of emigration resulted in lower net worth for MC immigrants compared to those from HK-T and white natives; although MC immigrants had higher net worth than Latino immigrants and native blacks, reflecting selection on education and income status.

In addition to studying total net worth, we explored differences among Chinese immigrants in their ownership of assets and accumulation of debt, the two components of net worth that have individual benefits (and potentially costs) for incorporation. We found that HK-T immigrants have relatively high propensities to have any assets or debts, and that those with assets, the values are particularly high. By contrast, MC immigrants have high propensities to own assets, and the values are higher than those owned by native whites and blacks but are not as high as the assets owned by HK-T immigrants. These high asset ownership rates are consistent with high rates of saving among Chinese in the home country, although our data are not able to speak to whether the immigrants we study are selected for high saving even relative to others in the home country. We found that debt levels vary by national origin as well: MC immigrants were less likely to own debts than HK-T immigrants and native whites and to have lower levels of debt, likely reflecting their lower levels of connection to the U.S. financial system and rates of business startup. Finally, we found important mediating effects of U.S. tenure and age that further underscore the differences among Chinese immigrants. That is, we found that as U.S. tenure increased, the wealth of all immigrants increased but the growth was particularly pronounced for MC immigrants, pushing them closer to the high levels of wealth seen among HK-T immigrants. Our age findings highlighted the degree to which Chinese immigrants share important cultural elements despite differences in economic advantage stemming from home country development: that is, we found an inverted U-shaped relationship between age and wealth for all Chinese immigrants that possible reflects a shared culture of close family relations that results in older relatives joining younger immigrants in the U.S. and also U.S. immigration policy that favors family reunification.

These findings provide important details about the role that within-group heterogeneity among Chinese immigrants may have for their adaptation and long-term class status stability and, thus, answer a growing call from immigration scholars to look more closely at differences within national-origin groups [12]. There is clear evidence that Chinese Americans have high education levels, at least in part because the highly-educated and those with educational aspirations are selected into immigration. Although educational attainment is highly correlated with other forms of attainment and incorporation, there is considerable variation by education on other measures of incorporation. There is little doubt that wealth levels are highly correlated across generations [70–72] and that wealth ownership contributes to long-term class stability [73–75]. Our work contributes to the literature on Chinese immigrants by documenting heterogeneity in wealth ownership among the immigrant generation and by studying the factors that contribute to wealth accumulation. Our findings suggest that long-term class stability is likely for Chinese immigrants; that said, our finding regarding the relatively low debt levels of MC immigrants may indicate that a portion of the Chinese immigrant community is not fully-integrated into the U.S. financial system. This pattern may reverse itself as relatively young MC immigrants complete their educations and enter into careers that may include business startup and real estate purchases. Future research could usefully study the debt acquisition of MC immigrants in coming years to clarify if and how these patterns change. Overall, however, the long-term economic prospects for Chinese immigrants appear bright.

The heterogeneity in Chinese immigrant wealth that we demonstrate also has implications for theoretical models of immigrant adaptation. We take a step back and investigate the economic adaptation of the immigrant generation, which creates the foundation and context for second-generation adaptation. Scholars debate whether and to what extent today’s new immigrants and their descendants are converging, or diverging, from the middle-class mean, and we build on this by showing that certain immigrants outperform the middle-class mean on wealth. Thus far, the debates have focused on parental education as one of the most critical indicators of second-generation mobility; our focus on wealth ownership also contributes to these debates by adding a more holistic economic outcome. While there is heterogeneity in wealth among Chinese immigrants, we find a pattern of accelerated economic adaptation, especially for those from HK-T, where wealth exceeds that of native-born whites and blacks, and nearly all other immigrant groups. However, Chinese immigrants as long-settled immigrants from the Mainland, and also those between the ages of 40–54 years old, exhibit a similar pattern of accelerated adaptation as their HK-T counterparts. These patterns reflect important within group differences and help to reformulate assimilation theories that typically homogenize immigrant groups and view adaptation as a process that occurs over time as the descendants of immigrant approximate the status of middle-class whites. Today’s immigrants do not always start off at the bottom, or even middle, tiers of the class structure and some may attain a level of economic adaptation in the immigrant generation that nearly approximates or surpasses that of long-settled whites. This pattern may have profound effects on the adaptation of their descendants who are slotted into an advantaged structural position relative to other immigrant groups, especially compared to Mexicans. The concept of accelerated economic adaptation via wealth accumulation may be applicable to other immigrant groups who are also hyperselected on income and education and who enter as citizens or legal residents. Our results also reveal grave disparities in wealth between today’s new immigrants.

This study provides important, new information about Chinese immigrants and assimilation theory, but it also leaves gaps that future research might usefully fill. As we discussed in our data section, the SIPP data have many strengths including the inclusion of large, nationally-representative samples of Chinese immigrants at multiple time periods with corresponding asset, debt, and demographic information. Unfortunately, the SIPP does not include longitudinal data necessary for studying wealth accumulation and other changes over time, nor does it include intergenerational information necessary for linking parents and children. The SIPP samples of Hong Kong and Taiwanese immigrants are small, preventing us from offering detailed comparisons across these two groups; and only citizens and legal residents are sampled, preventing us from exploring the effects of legal status on the processes we study. Future research would benefit from exploring Chinese American wealth accumulation using other datasets to understand whether unique traits of the SIPP limit or enhance our findings. The SIPP is also lacking a sample of high-wealth households that would make studying aggregate-level patterns of inequality possible. For example, estimating Gini coefficients or exploring the utility of models such as kinetic exchange models [76, 77, 78] would be useful for understanding aggregate-levels patterns of wealth inequality that result from the household-level patterns we study here. Future research could usefully expand in this direction.

Future research would also benefit from cross-national studies examining net worth, asset ownership, and debt within and between national-origin groups to demonstrate whether similar patterns are observed. Chinese-origin immigrants beyond the U.S. might exhibit different patterns of wealth accumulation due to variations in immigrant selectivity and opportunity structures in the host society. For example, the skills and educational distribution of Chinese migrants to the U.S. is heterogeneous, whereas Chinese immigrants to Spain tend to be low skilled and focused on small entrepreneurial endeavors [26]. Chinese enclaves in Spain are not as developed as they are in the U.S., Spain has a persistently high level of unemployment, and Chinese-origin youth have lower levels of educational attainment compared to any other ethnic group [79], potentially leading to patterns of wealth accumulation, and economic adaptation, that diverge from the U.S. case. On the other hand, Canada contains many highly skilled Chinese immigrants and developed ethnic enclaves, but Chinese immigrants in Canada often have much worse economic achievement than the general population [80].

There are also limitations in the scope of our study. For example, despite their relatively high levels of net worth, Chinese Americans do not have the political and economic power that typically accrues to the wealthy. Hence, accelerated economic adaptation does not necessarily translate into social and political integration [81], or access to traditional power structures in American society. Most evidence suggests that they remain underrepresented in politics and in executive positions in Fortune 500 companies. They also continue to face a “bamboo ceiling” in corporate America. We are unable to account for this paradox, but future research could usefully explore it. Finally, the observable differences in wealth between those from the Mainland and those from Hong Kong-Taiwan likely reflect the latter countries’ economic head start. However, the Chinese Savings Puzzle may be another explanatory factor. Future research could usefully explore these issues in more detail.

## References

[1] Hoeffel EM, Rastogi S, Kim MO, Shahid H. The Asian Population 2010. Washington D.C.: Census Bureau, 2012.

[2] Pew. The Rise of Asian Americans. Washington, DC: Pew Research Center, Pew Social and Demograhpic Trends, Pew Forum on Religion and Public Life, 2012.

[3] Walters NP, Trevelyan EN. The Newly Arrived Foreign-Born Population of the United States: 2010. Washington, DC: U.S. Census Bureau, 2011.

[4] Feliciano C. Unequal Origins: Immigrant Selection and the Education of the Second Generation. New York: LFC Scholarly Publishing; 2006.

[5] Lee J, Zhou M. The Asian American Achievement Paradox. New York: Russell Sage; 2015.

[6] Rumbaut RG. The Coming of the Second Generation: Immigration and Ethnic Mobility in Southern California. The Annals of the American Academy of Political and Social Science. 2008;620:196–236.

[7] Zhao X. The New Chinese America: Class, Economy, and Social Hierarchy. New Brunswich, NJ: Rutgers University Press; 2010.

[8] Zhou M. Contemporary Chinese America: Immigration, Ethnicity, and Community Transformation. Philadelphia PA: Temple University Press; 2009.

[9] Alba R, Jiménez TR, Marrow H. Mexican Americans as a Paradigm for Contemporary Intragroup Heterogeneity. Ethnic and Racial Studies. 2013;37:446–66.

[10] Agius Vallejo J. Barrios to Burbs: The Making of the Mexican-American Middle Class. Palo Alto: Stanford University Press; 2012.

[11] Bean FD, Stevens G. America's Newcomers and the Dynamics of Diversity. New York: Russell Sage Foundation; 2003.

[12] Waldinger R, Catron P. Modes of Incorporation: A Conceptual and Empirical Critique. Journal of Ethnic and Migration Studies. 2016;42:23–53.

[13] Bean F, Brown SK, Bachmeier JD. Parents Without Papers: The Progress and Pitfalls Mexican American Integration. New York: Russell Sage Foundation; 2015.

[14] Hao L. Color Lines, Country Lines: Race, Immigration, and Wealth Stratification in America. New York: Russell Sage Foundation; 2007.

[15] Zhou M. Revisiting Ethnic Entrepreneurship: Convergencies, Controversies, and Conceptual Advancements In: Portes A, DeWind J, editors. Rethinking Migration: New Theoretical and Empirical Perspectives. New York: Berghahn Books; 2007.

[16] Financial Times. China Income Inequality among World’s Worst https://www.ft.com/content/3c521faa-baa6-11e5-a7cc-280dfe875e28. January 2016.

[17] Xie Y, Goyette K. Asian Americans: A Demographic Portrait. Ann Arbor, MI: University of Michigan, Population Studies Center, Institute for Social Research, 2012.

[18] Alba R, Kasinitz P, Waters MC. The Kids are (Mostly) Alright: Second-Generation Assimilation. Social Forces. 2011;89:763–74.

[19] Haller W, Portes A, Lynch SM. Dreams Fulfilled, Dreams Shattered: Determinants of Segmented Assimilation in the Second Generation. Social Forces. 2011;89:733–62. 10.1353/sof.2011.0003 PMC3819936 24223437

[20] Keister LA, Agius Vallejo J, Borelli EP. Mexican American Mobility: An Exploration of Wealth Accumulation Trajectories. Social Forces. 2014;89:763–74.

[21] Waters MC J T. Assesing Immigrant Assimilation: New Empirical and Theoretical Challenges. Annual Review of Sociology. 2005;31:105–25.

[22] Portes A, Rumbaut RG. Legacies: The Story of the Immigrant Second Generation. Berkeley: University of California; 2001.

[23] Portes A, Zhou M. The New Second Generation: Segmented Assimilation and Its Variants. Annals of the American Academy of Political and Social Science. 1993;530:74–96.

[24] Zhou M. Growing up American: The Challenge Confronting Immigrant Children and Children of Immigrants. Annual Review of Sociology. 1997;23:63–95.

[25] Alba R, Foner N. Integration's Challenges and Opportunities in the Wealthy West. Journal of Ethnic and Migration Studies. 2016;42:3–22.

[26] Kasinitz P, Mollenkopf JH, Waters MC, Holdaway J. Inheriting the City: The Children of Immigrants Come of Age. New York: Russell Sage Foundation; 2008.

[27] Bean FD, Leach MA, Brown SK, Bachmeier JD, Hipp JR. The Educational Legacy of Unauthorized Migration: Comparisons Across U.S.-Immigrant Groups in How Parents' Status Affects Their Offspring. The International Migration Review. 2011;45(2):348–85. 22069771 10.1111/j.1747-7379.2011.00851.x

[28] Brown SK. Delayed Spatial Assimilation: Multigenerational Incorporation of the Mexican-origin Population in Los Angeles. City and Community. 2007;6:193–209.

[29] Park J, Myers D. Intergenerational Mobility in the Post-1965 Immigration Era: Estimates by an Immigrant Generation Cohort Method. Demography. 2010;47:369–92. 20608102 10.1353/dem.0.0105 PMC3000029

[30] Perlmann J. Italians Then, Mexicans Now: Immigrant Origins and Second-Generation Progress, 1890–2000. New York: Russell Sage; 2005.

[31] Lopez D, Stanton-Salazer R. Mexican Americans: A Second Generation at Risk In: Rumbaut RG, Portes A, editors. Ethnicities: Children of Immigrants in America. Berkeley: University of California Press; 2001.

[32] Jiménez TR. Replenished Ethnicity: Mexican Americans, Immigration, and Identity. Berkeley: University of California Press; 2010.

[33] Telles EE, Ortiz V. Generations of Exclusion: Mexican Americans, Assimilation, and Race. New York: Russell Sage Foundation; 2008.

[34] Fan CC. Chinese Americans: Immigration, Settlement, and Social Geography In: Ma LJC, Cartier C, editors. The Chinese Diaspora: Space, Place, Mobility,and Identity. Lanham, MD: Rowman and Littlefield; 2003.

[35] Zhou M, Tseng Y-F, Kim RY. Rethinking Residential Assimilation: The Case of a Chinese Ethnoburb in the San Gabriel Valley, California. Amerasia Journal. 2008;34:55–83.

[36] Zhou MaC, Guoxuan. Chinese Language Media in the United States: Immigration and Assimilation in American Life. Qualitative Sociology. 2002;25(3):419–41.

[37] Chua A, Rubenfeld J. The Triple Package: How Three Unlikely Traits Explain the Rise and Fall of Cultural Groups in America. New York: Penguin; 2015.

[38] Keister LA. Wealth in America: Trends in Wealth Inequality. New York: Cambridge University Press; 2000.

[39] Keister LA. Getting Rich: America's New Rich and How They Got that Way. New York: Cambridge University Press; 2005.

[40] Wolff EN. Recent Trends in Household Wealth, 1983–2009: The Irresistible Rise of Household Debt. Review of Economcs and Institutions. 2010;2:53–88.

[41] Zhou M. Chinatown: The Socioeconomic Potential of an Urban Enclave. Philadelphia, PA: Temple University Press; 1992.

[42] Haller AO, Portes A. Status Attainment Processes. Sociology of Education. 1973;46(1):51–91.

[43] Kerckhoff AC. The Status Attainment Process: Socialization or Allocation? Social Forces. 1976;55(2):368–81.

[44] Sorensen AB. A Model and a Metric for the Analysis of the Intragenerational Status Attainment Process. American Journal of Sociology. 1979;85(2):361–84.

[45] Campbell R, Henretta J. Status Claims and Status Attainment: The Determinants of Financial Well-Being. American Journal of Sociology. 1980;86:618–29.

[46] Keister LA, Lee HY. The One Percent: Top Incomes and Wealth in Sociological Research. Social Currents. 2014;1:13–24.

[47] Warren JR, Hauser R. Social Stratification across Three Generations: New Evidence from the Wisconsin Longitudinal Study. American Sociological Review. 1997;62:561–72.

[48] O'Rand AM, Krecker ML. Concepts of the Life Cycle: Their History, Meanings, and Uses in the Social Sciences. Annual Review of Sociology. 1990;16:241–62.

[49] Modigliani F. Life Cycle, Individual Thrift, and the Wealth of Nations. American Economic Review. 1986;76:297–313. 10.1126/science.234.4777.704 17744469

[50] Waters MC, Tran VC, Kasinitz P, Mollenkopf JH. Segmented Assimilation Revisited: Types of Acculturation and Socioeconomic Mobility in Young Adulthood. Ethnic and Racial Studies. 2010;33:1168–93. doi: 10.1080/01419871003624076 20543888 10.1080/01419871003624076 PMC2882294

[51] Alba R, Nee V. Remaking the American Mainstream: Assimilation and Contemporary Immigration. Cambridge, MA: Harvard University Press; 2003.

[52] Chi PSK, Kao C, Hsin P-L. Hong Kong and Taiwan Enterprises in Mainland China: Accelerators of Economic Transformation and Development? In: Cassel D, Herrmann-Pillath C, editors. The East, the West, and China's Growth: Challenge and Response. Baden-Baden, Germany: Nomos Verlagsgesellschaft; 1994.

[53] Hsiao FST, Hsiao M-CW. Economic Development of Taiwan: Early Experiences and the Pacific Trade Triangle. New York: World Scientific; 2015.

[54] Gold TB. State and Society in the Taiwan Miracle. New York: M.E. Sharpe; 1987.

[55] Hooper K, Batalova J. Hooper Kate and Batalova Jeanne. 2015 “Chinese Immigrants in the United States” Migration Policy Institute. Washington DC: Migration Policy Institute, 2015.

[56] Harbaugh RCsHSRTRoCRPaR, http://www.bus.indiana.edu/riharbau/harbaugh-chuxu.pdf. China's High Savings Rates. The Rise of China Revisited: Perception and Reality http://wwwbusindianaedu/riharbau/harbaugh-chuxupdf. 2004.

[57] Modigliani F, Cao SL. The Chinese Saving Puzzle and the Life-Cycle Hypothesis. Journal of Economic Literature. 2004;42:14–70.

[58] Yang DT, Junsen Zhang J, Zhou S. Why are Saving Rates fo High in China? In: Fan J, Morck R, editors. Capitalizing China. Chicago: University of Chicago Press; 2012 p. 249–78.

[59] Guthrie D. Dragon in a Three-piece Suit: the Emergence of Capitalism in China. Princeton, NJ: Princeton University Press; 1999.

[60] Lardy NR. China's Unfinished Economic Revolution. Washington, DC: The Brookings Institution Press; 1998.

[61] Naughton B. Growing Out of The Plan: Chinese Economic Reform, 1978–1993. New York: Cambridge University Press; 1996.

[62] Orrenius P, Zavodny M, Kerr E. Chinese Immigrants in the U.S. Labor Market: Effects of Post-Tiananmen Immigration Policy. International Migration Review. 2012;46:456–82.

[63] Kim EM. The Four Asian Tigers: Economic Development & the Global Political Economy. London: Emerald; 1999.

[64] Chow G. China's Economic Transformation. New York: Wiley-Blackwell; 2015.

[65] Naughton B. The Chinese Economy: Transitions and Growth. Boston: MIT Press; 2006.

[66] Hirschl TA, Rank MR. Homeownership Across the American Life Course: Estimating the Racial Divide. Race and Social Problems. 2010;2(3):125–36.

[67] Elder GH. The Life Course Paradigm: Social Change and Individual Development In: Moen P, Elder GH, Luscher K, editors. Examining Lives in Context: Perspectives on the Ecology of Human Development. Washington, DC: American Psychological Association; 1995 p. 101–40.

[68] Lan P-C. Subcontracting Filial Piety: Elder Care in Ethnic Chinese Immigrant Families in California. Journal of Family Issues. 2002;23:812–15.

[69] Treas J. Older Americans in the 1990s and Beyond. Population Bulletin. 1995;50:1–45.

[70] Mulligan CB. Parental Prioroties and Economic Inequality. Chicago: University of Chicago Press; 1997.

[71] Charles KK, Hurst E. The Correlation of Wealth across Generations. Journal of Political Economy. 2003;111:1155–82.

[72] Conley D, Glauber R. Wealth Mobility and Volatility in Black and White. Washington DC: Center for American Progress, 2008.

[73] Keister LA. The One Percent. The Annual Review of Sociology. 2014;40:347–67.

[74] Hansen MN. Self-Made Wealth or Family Wealth? Changes in Intergenerational Wealth Mobility. Social Forces. 2014;93:457–81.

[75] Khan SR. The Sociology of Elites. Annual Review of Sociology. 2012;38(1):361–77.

[76] Chatterjee A, Chakrabarti BK. Kinetic Exchange Models for Income and Wealth Distributions. European Physics Journal B. 2007;60:135–49.

[77] Pareschi L, Toscani G. Interacting Multi-agent Systems: Kinetic Equations and Monte Carlo Methods. New York: Oxford University Press; 2014.

[78] Chakrabarti BK, Chakraborti A, Chakravarty SR, Chatterjee A. Econophysics of Income and Wealth Distributions. New York: Cambridge University Press; 2013.

[79] Yiu J. Calibrated Ambitions: Low Educational Ambition as a Form of Strategic Adaptation Among Chinese Youth in Spain. International Migration Review. 2013;47:573–611.

[80] Wang S, Lo L. Chinese Immigrants in Canada: Their Changing Composition and Economic Performance. International Migration http://onlinelibrarywileycom/doi/101111/j1468-2435200500325x/abstract. 2005.

[81] Wong J, Ramakrishnan S. K, Lee T, Junn J. Asian American Political Participation: Emerging Constituents and Their Political Identities. New York: Rusell Sage Foundation; 2011.

